# The metabolic network model of primed/naive human embryonic stem cells underlines the importance of oxidation-reduction potential and tryptophan metabolism in primed pluripotency

**DOI:** 10.1186/s13578-019-0334-7

**Published:** 2019-08-29

**Authors:** Meisam Yousefi, Sayed-Amir Marashi, Ali Sharifi-Zarchi, Sara Taleahmad

**Affiliations:** 1 Department of Biotechnology, College of Science, University of Tehran, Tehran, Iran; 2 Department of Stem Cells and Developmental Biology, Cell Science Research Center, Royan Institute for Stem Cell Biology and Technology, ACECR, Tehran, Iran; 3 Department of Computer Engineering, Sharif University of Technology, Tehran, Iran

**Keywords:** Systems biology, Human embryonic stem cells, Tryptophan metabolism, Kynurenine metabolism, Genome-scale metabolic networks

## Abstract

**Background:**

Pluripotency is proposed to exist in two different stages: Naive and Primed. Conventional human pluripotent cells are essentially in the primed stage. In recent years, several protocols have claimed to generate naive human embryonic stem cells (hESCs). To the best of our knowledge, none of these protocols is currently recognized as the gold standard method. Furthermore, the consistency of the resulting cells from these diverse protocols at the molecular level is yet to be shown. Additionally, little is known about the principles that govern the metabolic differences between naive and primed pluripotency. In this work, using a computational approach, we tried to shed light on these basic issues.

**Results:**

We showed that, after batch effect removal, the transcriptome data of eight different protocols which supposedly produce naive hESCs are clustered consistently when compared to the primed ones. Next, by integrating transcriptomes of all hESCs obtained by these protocols, we reconstructed *p-hESCNet* and *n-hESCNet*, the first metabolic network models representing hESCs. By exploiting reporter metabolite analysis we showed that the status of NAD$^{+}$ and the metabolites involved in the TCA cycle are significantly altered between naive and primed hESCs. Furthermore, using flux variability analysis (FVA), the models showed that the kynurenine-mediated metabolism of tryptophan is remarkably downregulated in naive human pluripotent cells.

**Conclusion:**

The aim of the present paper is twofold. Firstly, our findings confirm the applicability of all these protocols for generating naive hESCs, due to their consistency at the transcriptome level. Secondly, we showed that in silico metabolic models of hESCs can be used to simulate the metabolic states of naive and primed pluripotency. Our models confirmed the OXPHOS activation in naive cells and showed that oxidation-reduction potential vary between naive and primed cells. Tryptophan metabolism is also outlined as a key pathway in primed pluripotency and the models suggest that decrements in the activity of this pathway might be an appropriate marker for naive pluripotency.

## Background

### Naive and primed pluripotent stem cells

Pluripotent stem cells are characterized by their self-renewal ability and their capacity to differentiate towards all three germ layers, namely ectoderm, mesoderm and endoderm [1]. Human pluripotent cells, whether isolated from blastocysts or reprogrammed from somatic cells, display distinguishable characteristics in comparison to mouse embryonic stem cells (mESCs). That is, they form flattened colonies, depend on FGF2 signaling in their culture media and are susceptible to single-cell trypsin passages [2]. It has also been shown that at the molecular level, there are major distinctions between the two cells, such as the activity of OCT4 enhancers and the status of X-chromosome inactivation [3, 4]. After the discovery of mouse epiblast stem cells, or mEpiSCs, which are pluripotent cells from mouse epiblast with high similarity to hESCs, it became clear that there are two distinct stages in pluripotency [5]. More specifically, cells like mEpiSCs and hESCs were proposed to be in the “primed” stage of pluripotency, while mESCs were in an earlier stage of development called the “naive” state [6].

Derivation of a naive-like hESC was reported in 2010 by Hanna et al. [7] for the first time. Since then, several groups have worked on developing more efficient protocols to produce cells which better resemble the naive state, both from embryonic stem cells and induced pluripotent stem cells (iPSCs) [8–12]. Whilst the initial protocols made efforts to convert conventional primed hESCs to naive cells, later attempts concentrated on deriving naive hESCs directly from blastocysts [13, 14] (Table 1). Recently, to compare the outcome of different proposed protocols for converting primed to naive hESCs, Warrier et al. [15] cultured the same cell lines under conditions suggested by three different protocols and sequenced their transcriptomes. They showed that these naive-like cells are more similar to each other compared to their primed counterparts.

**Table 1:** A comparison of protocols proposed to produce naive hESC

Protocol	Year	Origin	Growth factors	Inhibitors	Transgene expression
Hanna	2010	Primed hESC	TGF$\beta$ - LIF, BMP4	MEKi, GSKi, JNKi, P38i, PKCi, ROCKi	OCT4, SOX2, KLF4, KLF2
Gafni	2013	Primed hESC	bFGF, TGF$\beta$, LIF	MEKi, GSKi, FGFi, JAKi, ALKi, ROCKi	–
Ware	2014	Primed hESC	bFGF	MEKi, GSKi	–
Theunissen	2014	ICM	Activin, LIF	MEKi, GSKi, ROCKi, BRAFi, SRCi	KLF2, NANOG
Takashima	2014	Primed hESC	bFGF, LIF	MEKi, GSKi, PKCi	–
Valamehr	2014	Fibroblast (iPSC)	bFGF	MEKi, GSKi, ROCKi, ALKi	OCT4, SOX2
Duggal	2015	Primed hESC	bFGF, LIF	MEKi, GSKi, ROCKi	–
Guo	2016	ICM	bFGF, LIF	MEKi, GSKi, ROCKi, PKCi	–

Metabolism in naive and primed pluripotency has been investigated in mice. It was shown that EpiSCs almost exclusively rely on glycolysis, while mESCs are bivalent in their energy production, as they use both glycolysis and OXPHOS pathways [16]. Conventional primed hESCs and human iPSCs, like EpiSCs, have been shown to be essentially glycolytic [16, 17]. However, to the best of our knowledge, the metabolic states of human naive cells generated by each of the aforementioned protocols have not been studied comprehensively. Takashima et al. [12] investigated the metabolism of naive-like cells generated by their protocol. They showed that these cells, similar to mESCs, utilize OXPHOS along with glycolysis. They also showed that mitochondrial enzymes become activated in their naive-like cells. Later, Sperber et al. [18] showed that human naive cells also have greater oxygen consumption rate (OCR) than their primed counterpart. This shift in energy metabolism is suggested to affect the regulation of the epigenetic machinery, which in turn, is involved in the programming of the naive and primed pluripotency states [18, 19].

### A systems biology approach to the metabolism of naive and primed stem cells

A genome-scale metabolic network model (GEM) is a network of metabolites that are linked by potential reactions of cellular metabolism. Such a model is shown to be able to accurately predict metabolic phenotypes in silico [20]. Since the emergence of GEMs in the early 2000s, constraint-based modeling of metabolism using GEMs has been a powerful tool to study and predict cell metabolic behavior upon modifications and changes [21]. Using GEMs, one can quantitatively predict fluxes running through each reaction and pathway of a cell [22], especially in unicellular organisms. The first human GEM, Recon 1, was reconstructed in 2007, which included all known reactions and metabolites over all human tissues [23]. Contrary to unicellular organisms, each cell type in the human body employs its own particular set of reactions. Context-specific metabolic networks are metabolic sub-models derived from the generic human metabolic network, which identify (potentially) active reactions based on ‘omics’-scale data. Recently, Chandrasekaran et al. generated the first metabolic network model representing pluripotency by analyzing time-series metabolomics data of naive and primed mouse pluripotent cells [24, 25].

None of the current protocols to generate naive hESC is globally recognized as the gold standard method. This problem could raise the question about the rationality of generalizing the specific findings of one study using one particular protocol to all naive cells. Here, by comparing the transcriptomic profiles of eight different protocols generating naive hESCs, we show that the general expression patterns of the naive cells from these protocols are similar to each other and can be distinguished from their conventional primed counterparts. Then, we present “*hESCNet*”, the first metabolic network model specific for human embryonic stem cells. Using this model, we compared the overall metabolic states of the primed and naive hESCs. We investigated the active metabolic pathways in primed and naive cells and we showed that OXPHOS and tryptophan (Trp) metabolism is crucial for naive and primed cells, respectively.

## Results

### Data collection, batch effect removal and heterogeneity analysis

**Fig. 1:**
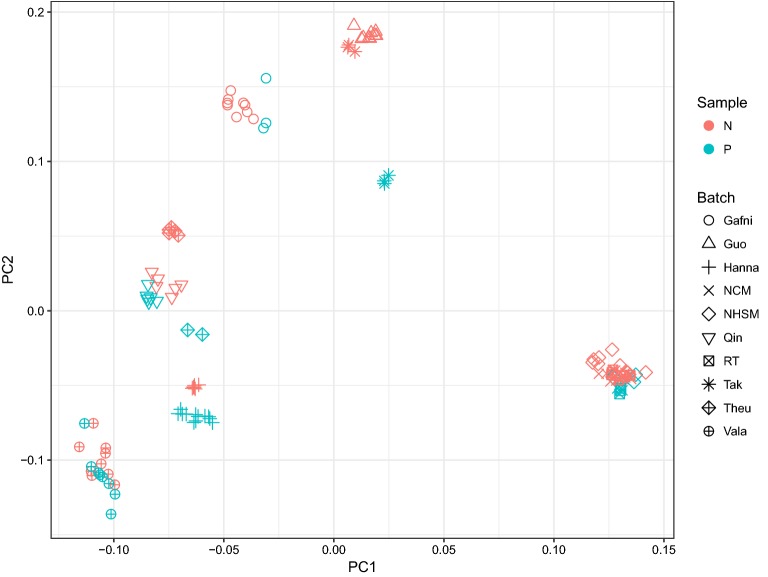
PCA of transcriptome profiles of different protocols: cells are clustered by their experiment origin

**Fig. 2:**
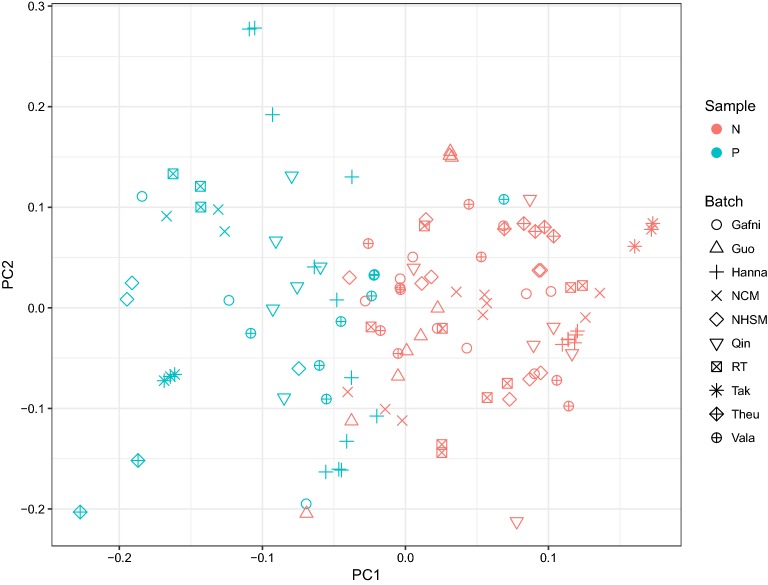
PCA of transcriptome profiles of different protocols after using ComBat: naive and primed pluripotent cells are separated

**Fig. 3:**
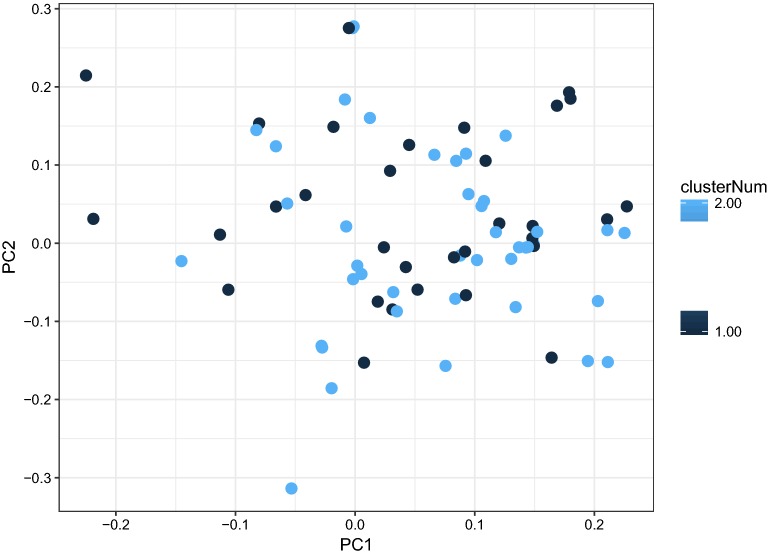
PCA of naive cells transcriptome profiles after *k*-means clustering analysis: arbitrary dispersion of the two clusters (dark and light blue) depict that no major heterogeneity exists among naive cells

To compare the expression patterns in naive and primed pluripotency, we obtained raw transcriptome data of Hanna et al. [7], Gafni et al. [9] (NHSM), Valamehr et al. [8], Theunissen et al. [13], and Takashima et al. [12]. We also included the data of Warrier et al. [15] which has employed NHSM [9], NCM [11] and RT [10] protocols to generate naive cells. After the initial transcriptome data analysis and normalization, we performed principal components analysis (PCA) to gain insight about the status of naive vs. primed cell data. By plotting the first two components, we observed a clear batch effect in the data, as cells being clustered together by their experiment origin rather than their pluripotency status (Fig. 1). Notably, after removing batch effects using ComBat function, naive and primed cells from different studies were clustered together (Fig. 2). This observation confirms that the overall expression profiles of different (supposed) naive cells are generally similar to each other at the transcriptome level. To ensure that there is no major heterogeneity among our naive cells, after batch effect removal, we performed *k*-means clustering on the data. By enforcing all naive cells to cluster into two groups, no significant grouping was obtained, which shows that there was no obvious heterogeneity in the data (Fig. 3). Therefore, this relative “homogeneity” paves the way to rely on all these protocols.

### Evaluation of gene expression data after batch effect removal

**Fig. 4:**
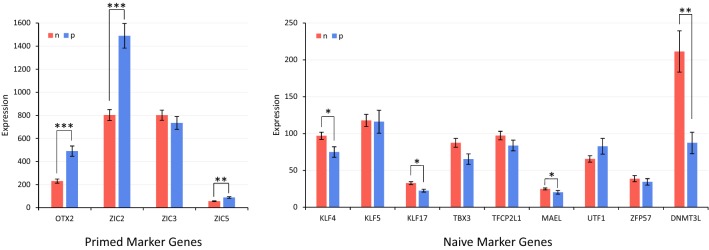
Expression pattern of genes previously reported as naive or primed markers: *: adjusted *p*-value < 10$^{-2}$, **: adjusted *p*-value < 10$^{-4}$, ***: adjusted *p*-value < 10$^{-6}$. (Genes whose *p*-values are not indicated were non-significant)

In our dataset, we examined the expression patterns of the previously reported biomarkers of naive and primed pluripotency [26]. When samples from different studies with different expression ranges are considered, even a normalization step may adversely impact the quality of (and introduce biases in) the data, let alone the batch effect removal procedure. Therefore, one might expect some irrelevant genes to have significant *p*-values after differential gene expression analysis. Nevertheless, in our study, all those biomarkers which had a significant differential gene expression showed appropriate expression in the state they were representing (Fig. 4). Notably, DNMT3L which had one of the most significant differential expressions was reported to regulate naive cells epigenome profile [18].

For the set of differentially expressed genes, we then performed a KEGG Pathways enrichment analysis. Interestingly, among down-regulated genes in naive cells, pathways related to cell adhesion such as “ECM receptor interaction”, “cell adhesion molecules” and “tight junction”, appeared in the most significant enriched pathways (Table 2). This observation is consistent with previous studies investigating cell adhesion in mESCs [27]. The complete sets of enrichment analysis results are provided in Additional file 1: Tables S4 and S5.

**Table 2:** Results of KEGG pathways enrichment analysis for down-regulated genes in naive hESCs

KEGG pathway terms	Size	FDR *q*-value
Axon guidance	119	0.009
ECM receptor interaction	72	0.018
Cell adhesion molecules (CAMs)	95	0.020
Tight junction	110	0.032
Leukocyte transendothelial migration	96	0.033
Antigen processing and presentation	41	0.040

Only statistically significant pathways are shown here

### Metabolic network model reconstruction

We used CORDA2 [28] to reconstruct the hESC-specific metabolic network based on the generic human metabolic network model. This model, which will be referred to as (*hESCNet*), is $\sim 44\%$ smaller than the generic model based on the number of reactions, which is acceptable considering the non-parsimonious approach of the CORDA algorithm (Additional file 2: hESCNet_model). The main characteristics of *hESCNet* are shown in Table 3. To make model prepared for FVA, we added a biomass reaction [29] as the objective function to *hESCNet*. Details about biomass constituents and their coefficients are provided in Additional file 1: Table S3.

**Table 3:** The characteristics of *hESCNet* model

	Recon 2.2	hESCNet
Metabolites	5324	2483
Reactions	7785	4414
Genes	1675	1420

### Reporter metabolite analysis

**Fig. 5:**
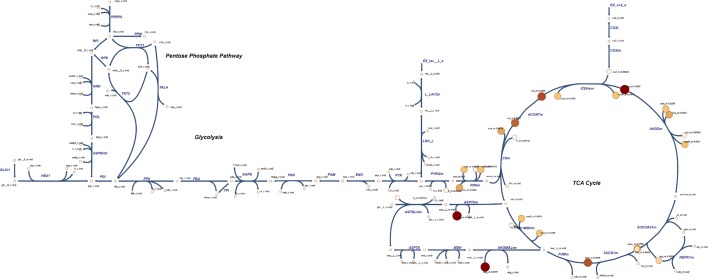
Reporter metabolite analysis results in central carbon metabolism: genes associated to TCA cycle reactions were among the most altered genes between human primed and naive pluripotency. Numbers show 1-*p*-value for reporter metabolites

Reporter metabolite analysis algorithm aims to find those metabolites in a network around which the most significant transcriptional changes has occurred [30]. We integrated the *p*-values of differentially-expressed genes to *hESCNet* in order to obtain the list of reporter metabolites (Additional file 1: Table S6). We then mapped the reporter metabolites with significant *p*-values to our metabolic network. Notably, metabolites associated with TCA cycle were mostly found to be among the reporter metabolites (Fig. 5). This observaion confirms the essentiality of the “dual energy metabolism” in naive cells [16]. Nicotinamide adenine dinucleotide (NAD$^+$) was also a reporter metabolite, which has a fundamental role in adjusting the oxidation-reduction potential of the cell. Although not extensively investigated, the NAD$^+$/NADH redox state has been proposed to have a role in the state of cell pluripotency [31].

### Flux variability analysis

To compare naive and primed metabolism quantitatively, we decided to have a separate metabolic network for each pluripotency stage. Like previous studies [32, 33], highly-downregulated genes in naive cell were removed from *hESCNet* to acquire a naive model (*n-hESCNet*), highly-downregulated genes in primed cell were removed to acquire a primed one (*p-hESCNet*). Next, we performed flux variability analysis (FVA) on these models to compare fluxes running through each reaction in naive and primed models. To achieve more accurate results, we also constrained model exchange reactions according to the growth medium composition (Additional file 1: Table S9).

**Fig. 6:**
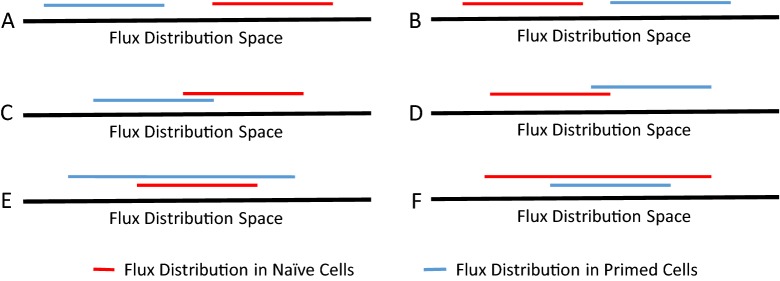
Major possibilities for each reaction after FVA: other possible distributions not included

**Fig. 7:**
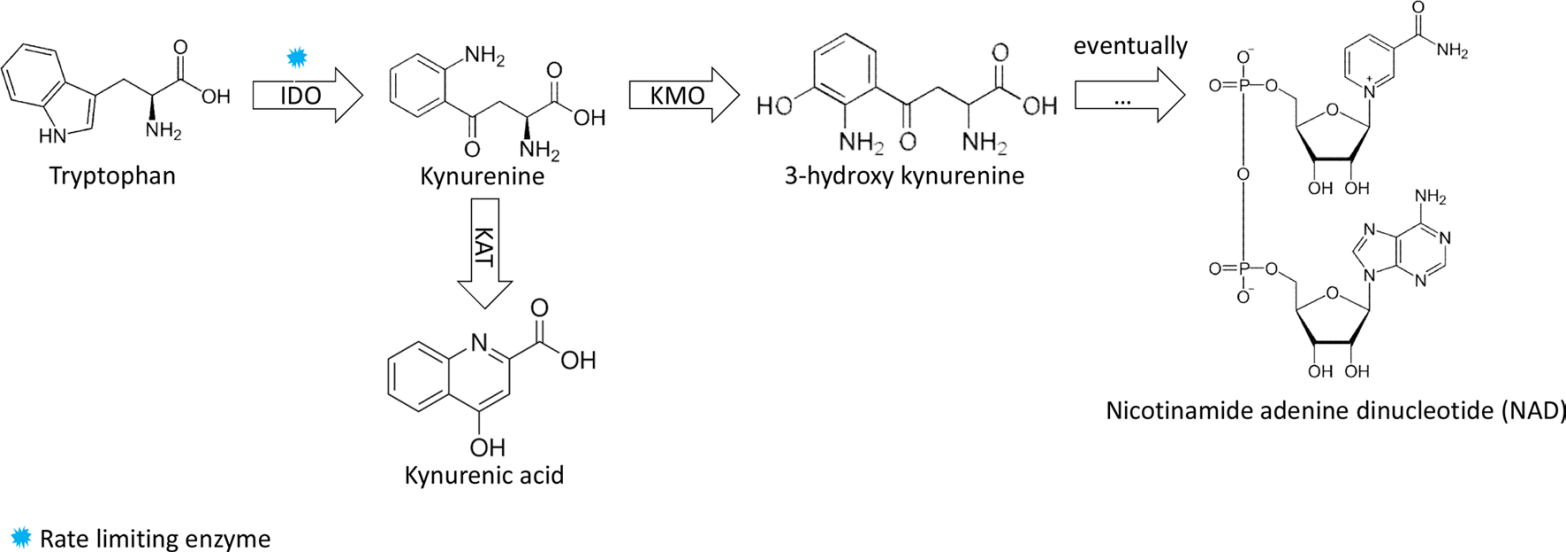
Schematic kyneurenine-mediated catabolism of tryptophan pathway

By comparing flux distributions in naive and primed models, six major possibilities can occur for a reaction (Fig. 6, [32]). Reactions in statuses “A” and “C” were considered to be upregulated in naive cells, while reactions in statuses “B” and “D” were considered upregulated in primed cells. The complete list of reaction statuses is provided in Additional file 1: Table S8. Genes associated with these upregulated and downregulated reactions were used for KEGG pathway enrichment analysis (see Table 4). Our results suggest that the most significantly downregulated metabolic pathway in naive pluripotent cells is the metabolism of tryptophan, an amino acid which is recognized for its pivotal role in cancer [34]. Among all the metabolic pathways associated with tryptophan, we observed that most of the genes involved in the kynurenine pathway are downregulated in naive cells. IDO1, which is the rate-limiting enzyme in this pathway, is also in this list. An overall diagram of the pathway is shown in (Fig. 7).

**Table 4:** KEGG pathways enrichment analysis using DAVID

KEGG pathway terms^a^	Size	*p*-value	Benjamini adjusted *p*-value
Tryptophan metabolism	6	3.02E−08	5.13E−07
Cysteine and methionine metabolism	4	1.00E−04	0.001136
Fatty acid degradation	4	1.36E−04	0.001152
Fatty acid metabolism	4	2.02E−04	0.001376
Valine, leucine and isoleucine degradation	3	0.005821	0.032542

^a^The term “Metabolic pathways” is excluded from the table due to its triviality. Only pathways with significant *p*-values are shown

## Discussion

For years, the state of stem cell metabolism was considered as a byproduct, rather than the cause of the cell pluripotency status . However, emerging studies emphasize the importance of metabolism as a driver of regulatory mechanisms to control lineage commitments and self-renewal [18, 35, 36]. Naive pluripotent stem cells are no exception to this scenario. Relatively little attention was paid to a systematic evaluation of metabolic changes during naive-to-primed conversion [37], while the existence of multiple methods for generating naive hESCs has complicated these kinds of investigations.

In this work, using a meta-analysis approach, we demonstrated that different naive cells generated by different protocols and studied by different transcriptomic platforms exhibit similar molecular characteristics when it comes to metabolism. To this end, after batch effect removal of transcriptome data, we found a clear distinction between naive and primed hESCs. Moreover, one could observe that the samples which appeared in the border of naive-primed cell data belong to earlier protocols (including Hanna et al. and Valamehr et al.). We also showed that despite different origins, naive cells obtained by different protocols do not display an apparent heterogeneity among themselves. This observation emphasizes that all the aforementioned protocols describe similar cells.

Tryptophan metabolism essentiality has been previously studied in pluripotency. One of the main tryptophan metabolic pathways goes through kynurenine, an aromatic non-proteinogenic amino acid, which eventually results in NAD$^+$ production. Roles of kynurenine pathway in adult stem cells, including neural stem cells and hematopoetic stem cells, has been studied before [38]. However, the possible role of this pathway in pluripotency has remained unexplored. Using mass spectrometry, kynurenine levels has been reported to be significantly increased (by 27 folds) in primed human embryonic cells in comparison to embryonal carcinoma cells [39]. Interestingly, recent investigations on tumors, have reported kynurenine’s impact on signaling cascades such as Wnt, Notch and PI3K, which are fundamental signaling pathways for pluripotency as well [40, 41]. We also observed that IDO1, a key enzyme in tryptophan degradation through kynurenine, was downregulated in all the naive cells (Additional file 1: Table S2), which further underlines the importance of kynurenine pathway in primed pluripotency. It has previously been shown that blockade of IDO1 would results in $\beta$-catenin stabilization in the cytoplasm which is critical in pluripotency [42]. IDO has also been reported to regulate mTOR pathway [43]. The outcome of our computational model is in consistency with Sperber et al. study, indicating kynurenine pathway as a markedly up-regulated pathway in primed hESCs. Based on the results of our metabolic network model of human pluripotency and previous studies, we suggest that there is a great potential in kynurenine catabolism pathway to be investigated in pluripotency. Furthermore, we propose that kynurenine metabolism could be an appropriate candidate marker of primed pluripotent cells against naive ones. we also showed that NAD$^+$ is a reporter metabolite in naive human pluripotency and considering that NAD$^+$ is the final product of kynurenine pathway, we suggest that the oxidation-reduction state and especially NAD$^+$/NADH balance are proper candidates to be investigated in naive and primed pluripotency.

In this work, we utilized computational models of cell metabolism to study hESCs and naive/primed pluripotency. Although our results are consistent with the previous wet-lab reports, one should keep in mind the limitations of implementing computational models in cell biology research. Current metabolic models do not perfectly represent cell metabolism due to our inadequate knowledge of cell metabolism and its dynamism. Furthermore, the outcome of each analysis may depend on the chosen algorithms for reconstruction and analysis of the context-specific models. Therefore, to further validate the roles of our proposed candidate pathways in pluripotency, one should thoroughly investigate their roles in vitro [44, 45].

## Conclusion

There are several protocols which have the claim to generate naive hESCs. Despite a few attempts on some of these naive cells, no comprehensive study has fully compared cells generated by these diverse protocols. In this work, utilizing the transcriptome data of eight protocols producing naive human pluripotent cells, we showed that the general expression profiles of naive and primed hESCs are distinctive to each other and no apparent heterogeneity exist among these naive cells.

Besides the attempts that have taken place to compare different naive cells so far (i.e., Warrier et al.), in most of the papers which introduce such a new protocol, the resulting naive cells are also compared to those of the previous protocols. There also have been some studies comparing these protocols to human preimplantation embryo cells [18, 46]. In our work, we compared active pathways in naive and primed cells. However, one may also think of applying a similar strategy to study the potential “among-protocol” differences at the systems level. We believe that with the available data, due to the heterogeneity of the data sources, this task might not be easily achievable. The best way to perform this task is to utilize an approach similar to Warrier’s study, where different protocols are imposed to the same naive cells in the same laboratory and the same platform is used to obtain omics data, and then, use these data to reconstruct and analyze cell-specific metabolic network for each protocol [15].

Using the transcriptome data, we also reconstructed *hESCNet*, the first metabolic network model representing hESCs. This model confirmed the dual energy metabolism of naive pluripotent cells and also proposed that NAD$^+$/NADH balance is likely to have a role in naive pluripotency. By extracting *p-hESCNet* and *n-hESCNet* models for primed and naive cells respectively, we also showed that metabolic flux distribution of kynurenine-mediated catabolism of tryptophan significantly differs between naive and primed state. This work, paves the way for future studies on naive pluripotency in human, and proposes that oxidation-reduction potential of cell and tryptophan metabolism are proper candidates to be further investigated in this context.

## Methods

### Transcriptome data collection and analysis

Expression profiles of studies used in this article were obtained from their repository web pages at GEO under accession numbers of GSE59435, GSE50868, GSE69200, GSE46872, GSE21222 and PRJNA356255, and ArrayExpress under accession numbers of E-MTAB-2857 and E-MTAB-4461. In case of RNA-seq data, Trimmomatic software was used to trim low quality reads [47]. Further details about these data and samples are provided in (Table 5).

**Table 5:** Details about the transcriptome data used in this work. Overall gene number is the number of mutual genes between all the transcriptome data

Protocol	Year	Data accession	Technique	Naive samples	Primed samples	Genes
Hanna et al.	2010	GSE21222	Microarray	6	12	21754
Gafni et al.	2013	GSE46872	Microarray	9	3	20002
Valamehr et al.	2014	GSE50868	Microarray	9	8	21754
Theunissen et al.	2014	GSE59435	Microarray	5	2	20015
Takashima et al.	2014	E-MTAB-2857	RNA-seq	3	3	15950
Qin et al.	2016	GSE69200	Microarray	6	6	20756
Guo et al.	2016	E-MTAB-4461	RNA-seq	9	NA^a^	58726
Warrier et al.	2017	PRJNA356255	RNA-seq	27	9	23375
Overall				74	43	14352

^a^Guo et al. used the primed hESC samples data from Takashima et al., a previous study by the same research group

RNA-seq transcriptome data were aligned against human hg38 reference genome using HISAT2 [48]. The obtained SAM files were sorted using SAMtools and raw count tables were generated by HTSeq [49, 50]. Normalization and differential gene expression analysis were performed by DESeq2 and Limma packages in R [51–53]. Microarray transcriptome data were analyzed by Limma package. All gene expression profiles were merged and batch effects were removed, in an unsupervised manner, by ComBat function of the SVA package in R [54]. Expression data table after batch effect removal is provided in (Additional file 1: Table S1). Enrichment analysis for KEGG pathways were performed by Gene Set Enrichment Analysis (GSEA) v2.0 [55, 56]. Graphs were produced by ggplot2 package in R and Excel [57].

### Metabolic network reconstruction

Human generic metabolic network model (Recon 2.2) was obtained from Swainston et al. [58]. Several algorithms had been developed to derive context-specific metabolic network models from the genome-scale ones [59–63]. CORDA2 algorithm was chosen to reconstruct the context specific network model in the COBRA toolbox v3.0 in Matlab 2017b [28, 63, 64]. In order to reconstruct the network, CORDA needs three reaction sets: (a) High confidence reactions (i.e., those reactions whose associated enzyme(s) are highly expressed); (b) Medium confidence reactions (i.e., those reactions whose associated enzyme(s) are moderately expressed); and (c) Negative confidence reactions (i.e., those reactions whose associated enzyme(s) are lowly expressed). Using mapExpressionToReactions function, we mapped our transcriptome data to Recon 2.2 reactions. Non-metabolic genes and genes not present in Recon 2.2 model were treated as “not expressed” as well. Top 10 percent of all reactions with the greatest expression levels were treated as High confidence based on the results of a systematic review on context specific metabolic network reconstruction [29]. Similarly, 10 percent of all reactions with the smallest expression levels were treated as Negative confidence reactions. In order to set exchange reactions boundaries, composition of cell culture medium was determined based on Sigma-Aldrich website for Dulbecco’s Modified Eagle’s Medium (DMEM) Additional file 1: Table S9.

### Reporter metabolite analysis

Differentially expressed genes (DEGs) and their adjusted *p*-values were computed by DESeq2 package in R (Additional file 1: Table S2). The reporter metabolite analysis was performed by the reporterMetabolites function in RAVEN toolbox. Metabolites with a significant adjusted *p*-value (< 0.05) were selected and transported to Escher online (https://escher.github.io) for illustration [65].

### Flux variability analysis (FVA)

In order to generate distinct networks for primed and naive cells from *hESCNet*, we removed highly downregulated genes in each pluripotency stage to obtain two distinct models. To avoid the potential bias stemming from sensitivity of FVA results to removal *p*-value thresholds, DEGs were selected by three different fold-change thresholds: (a) logFC > 1.00; (b) logFC > 0.85; and (c) logFC > 0.7 (with adjusted *p*-value < 0.05 for all). By setting the lower- and upper-bounds of their associated reactions to zero, we practically removed these gene sets in *hESCNet*, resulting in three models representing naive hESC and three models representing primed hESC. Flux variability analysis (FVA) was performed using the fluxVariability function in COBRA toolbox. The resulting flux distibution sets computed for each reaction were compared between naive and primed cells. Genes associated with those selected active reactions were obtained using findGenesFromRxns function in COBRA toolbox. Pathway enrichment analysis were performed using the Database for Annotation, Visualization and Integrated Discovery (DAVID) v6.8 [66, 67].

## Supplementary information

**Additional file 1: Table S1.** Gene expression data. **Table S2.** Differentially expressed genes list. **Table S3.** Biomass reaction. **Table S4.** KEGG pathways down-regulated in naive cells. **Table S5.** KEGG pathways up-regulated in naive cells. **Table S6.** Reporter metabolites list. **Table S7.** List of reactions up/down-regulated in naive cells to be knocked-out in hESCNet. **Table S8.** FVA outcome comparing p-hESCNet and n-hESCNet. **Table S9.** Exchange reactions boundaries.

**Additional file 2.** hESCNet_model.

## Data Availability

All data generated or analyzed during this study are included in this published article.
